# Construction of Simplified Microbial Consortia to Degrade Recalcitrant Materials Based on Enrichment and Dilution-to-Extinction Cultures

**DOI:** 10.3389/fmicb.2019.03010

**Published:** 2020-01-10

**Authors:** Dingrong Kang, Samuel Jacquiod, Jakob Herschend, Shaodong Wei, Joseph Nesme, Søren J. Sørensen

**Affiliations:** ^1^Section of Microbiology, Department of Biology, University of Copenhagen, Copenhagen, Denmark; ^2^Agroécologie, AgroSup Dijon, INRAE Centre Dijon, Université de Bourgogne, Université de Bourgogne Franche-Comté, Besançon, France

**Keywords:** simplified microbial consortia, biodegradation, enrichment cultivation, dilution-to-extinction, recalcitrant materials

## Abstract

The capacity of microbes to degrade recalcitrant materials has been extensively explored for environmental remediation and industrial production. Significant achievements have been made with single strains, but focus is now going toward the use of microbial consortia owning to their functional stability and efficiency. However, assembly of simplified microbial consortia (SMC) from complex environmental communities is still far from trivial due to large diversity and the effect of biotic interactions. Here we propose a strategy, based on enrichment and dilution-to-extinction cultures, to construct SMC with reduced diversity for degradation of keratinous materials. Serial dilutions were performed on a keratinolytic microbial consortium pre-enriched from a soil sample, monitoring the dilution effect on community growth and enzymatic activities. An appropriate dilution regime (10^–9^) was selected to construct a SMC library from the enriched microbial consortium. Further sequencing analysis and keratinolytic activity assays demonstrated that obtained SMC displayed actual reduced microbial diversity, together with various taxonomic composition, and biodegradation capabilities. More importantly, several SMC possessed equivalent levels of keratinolytic efficiency compared to the initial consortium, showing that simplification can be achieved without loss of function and efficiency. This methodology is also applicable to other types of recalcitrant material degradation involving microbial consortia, thus considerably broadening its application scope.

## Introduction

Microbes hold promising application potential to raise the efficiency of bioprocesses when dealing with substances that are resistant to decomposition (Subashchandrabose et al., 2011; Shong et al., 2012). A large number of microorganisms have been isolated based on their ability to degrade recalcitrant materials such as lignocellulose and polyurethanes (Brown and Chang, 2014; Mahajan and Gupta, 2015). In many cases of degradation efficiency, microbial consortia have been found superior when compared to single strains (Mikesková et al., 2012). For example, novel thermophilic consortia of *Brevibacillus* spp. and *Aneurinibacillus* sp. have been isolated from the environment to enhance polymer degradation (Skariyachan et al., 2018). Two approaches exist to obtain microbial consortia involving either (i) a synthetic assembly from scratch by combining several isolated strains (Puentes-Téllez and Salles, 2018), or (ii) obtainment of complex microbial communities from environmental samples (Skariyachan et al., 2017). For the later, enrichment process is often used to get the desired microbial consortia (Luo et al., 2016; Burniol-Figols et al., 2018; Kang et al., 2018). For instance, a termite gut-derived consortium showing a high xylanase activity was enriched on raw wheat straw as the sole carbon source, which was able to transform lignocellulose into carboxylates under anaerobic conditions (Lazuka et al., 2018). Yet, relatively high diversity levels are still observed despite the use of enrichment steps when working from environmental samples (Kang et al., 2018), likely due to the high functional redundancy observed in environmental microbial communities, being a key asset of their functional stability (Shade et al., 2012; Awasthi et al., 2014). This intrinsic diversity may stand as a bottleneck in attempts to move forward to practical application due to (i) potential negative correlation with efficiency (Banerjee et al., 2016), (ii) real microbial cheaters whose presence has no impacts on degradation, (iii) security threats posed by the presence of known or unknown pathogens, and (iv) risks of losing the properties of interest if supported by rare taxa.

Utilization of microbial consortia with less complexity, but equal efficiency, can lead to more controlled and optimized industrial processes (Puentes-Téllez and Salles, 2018). For instance, a large proportion of functional genes were remarkably altered and the efficiency of diesel biodegradation was increased by reducing the biodiversity of a microbial community from diesel-contaminated soils (Jung et al., 2016). Therefore, it is crucial to find reliable strategies to narrow down the diversity toward optimized microbial consortia gained from environmental samples. A reductive-screening approach was applied to construct effective minimal microbial consortia for lignocellulose degradation based on different metabolic functional groups (Puentes-Téllez and Salles, 2018). Additionally, artificial selection approaches (dilution, toxicity, and heat) have been also employed to obtain bacterial consortia (Lee et al., 2013). Among them, dilution-to-extinction has already proven its efficiency for obtaining functional microbial consortia from seawater and rumen liquor (Ho et al., 2012b; Hoefman et al., 2012; Sosa et al., 2015). Dilution-to-extinction is expected to provide more advantages compared to conventional isolation and assembly as it (i) generates many microbial combinations ready to be screened, (ii) includes strains from the initial microbial pool that might be lost due to cultivation/isolation biases, and (iii) ensures that all microbes are physically present and interacting spontaneously (Roger et al., 2016).

Keratins are recalcitrant fibrous materials with cross-linked components, representing the most abundant proteins in epithelial cells (Coulombe and Omary, 2002). They are estimated to have considerable economic value after biodegradation (Korniłłowicz-Kowalska and Bohacz, 2011). An efficient keratinolytic microbial consortium (KMCG6) was previously enriched from an environmental sample through cultivation in keratin medium (Kang et al., 2018). Despite reducing the microbial diversity during the enrichment process, KMCG6 still included several OTUs scattered amongst seven bacterial genera. This study aims to gain simplified microbial consortia (SMC) with fewer species but similar keratinolytic activity from this original consortium KMCG6. An adapted concept of dilution-to-extinction cultures was applied to gain various SMC from one optimal dilution level. This strategy integrating enrichment and dilution-to-extinction cultures is expected to be an effective way to obtain SMC for recalcitrant material biodegradation.

## Materials and Methods

### Substrate and Medium Preparation

About 20 kg of mixed α-keratin materials (raw bristles and hooves) were collected from a Danish Crown slaughterhouse (Bragesvej, Denmark) on March 22, 2014. The fresh materials were thoroughly washed with tap water and cut to about 2 mm in diameter mechanically by Daka Sarval (Løsning, Denmark). Subsequently, a sterilization process combining steam heating and pressure was applied (150°C, six bars, 20 min). The materials were further processed by drying and milling before use. The initial nutrient contents of processed keratin materials consist of more than 90% crude protein (Kang et al., 2018). Keratin medium (KM) was prepared with 1% keratinous material with mineral salt medium (0.5 g/L of NH_4_Cl, 0.5 g/L of NaCl, 0.3 g/L of K_2_HPO_4_, 0.4 g/L of KH_2_PO_4_, 0.1 g/L of MgCl_2_ 6H_2_O) (Bertsch and Coello, 2005), with keratin being the sole carbon source. KM was sterilized by autoclaving (120°C, 21 min).

### KMCG6 Obtainment and Serial Dilutions

**FIGURE 1:**
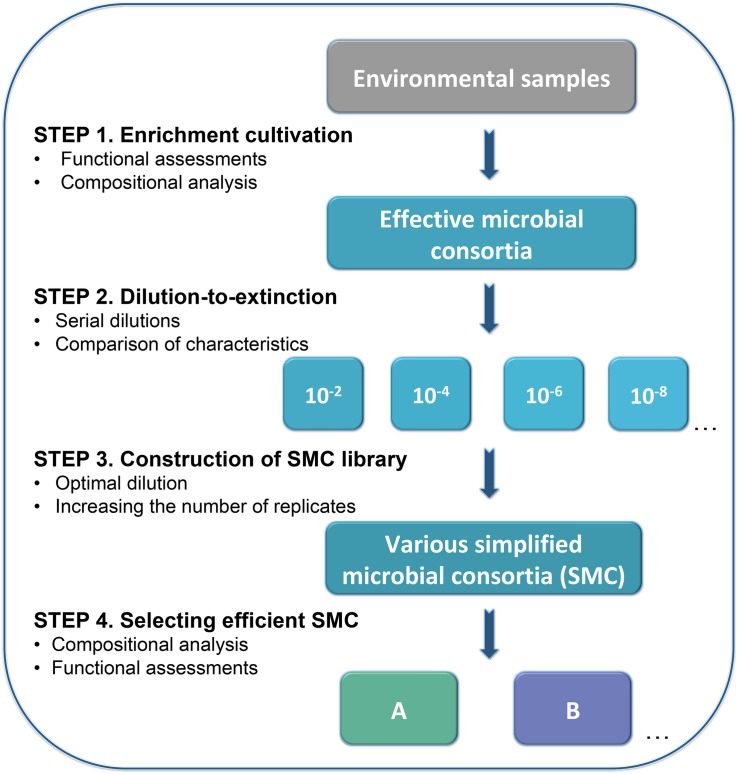
Workflow of enrichment and dilution-to-extinction cultures to select simplified microbial consortia (SMC) for keratin degradation. This workflow includes four steps: (1) Enrichment for the desired traits e.g., keratinolytic activity by selection in keratin medium, where keratin is the sole carbon source. This process was evaluated by functional assessments (cell density, enzymes activity, and ratio of the residual substrate) and compositional analysis. (2) Serial dilutions were conducted to the enriched effective microbial consortia. Six dilutions were prepared, from dilution 10^–2^ to 10^–10^ with 24 replicates. The dissimilarity between dilutions was evaluated by Euclidean distance calculation based on functional assessment criteria. (3) Library construction was done from the dilution offering the optimal dissimilarity among replicates. Dilution 10^–9^ was selected to construct the SMC library in this case. (4) Selection of the most promising SMC is based on functional and compositional characterization.

The microbial consortium “KMCG6” was obtained from a river bank soil sample after serial enrichments in successive generations of batch cultivation (batch culture “Keratin Microbial Consortia Generation 6”, resulting in consortium “KMCG6”) (Kang et al., 2018). KMCG6 was cultured overnight in LB medium with shaking (250 rpm, 24°C) until the exponential phase with OD about 1.0. Despite not being the medium used for selection, LB medium was chosen as a trade-off for guaranteed efficient propagation of this particular consortium after cryopreservation as it (i) contains peptone, thus ensuring proteolytic activity, and (ii) ensures sufficient cell density within a reasonable time from all dilutions. LB has successfully been used previously to reactivate KMCG6 from cryopreservation with maintained keratinolytic activity (Kang et al., 2018). Subsequently, serial dilutions (10^–2^, 10^–4^, 10^–6^, 10^–8^, 10^–9^, and 10^–10^) were performed to the cultured KMCG6 (Figure 1). 24 wells of a 96-well plate were inoculated with 200 μL suspension from each dilution, resulting in 144 diluted microbial consortia from six different dilutions. 96-well plates were incubated 1 to 2 days (250 rpm, 24°C) with equal cell density, following 150 μL culture from each well was inoculated into 1.5 mL KM growing in 24-well plates separately. Functional assessments (cell density and enzyme activity assays) of diluted microbial consoria were performed after 3 days of incubation (250 rpm, 24°C), which was shown to be the activity peak according to our previous work (Kang et al., 2018).

### Construction of the Microbial Consortia Library

The dilution 10^–9^ was selected as being the optimal dilution level after evaluation of the functional assessments described above. Subsequently, a SMC library was constructed at this particular dilution level with a larger number of replicates (Figure 1). In total, a library of 96 SMC was created at dilution 10^–9^ from KMCG6. 18 SMC with different characteristics in terms of cell density and protease activity were further selected from the library (Supplementary Figure S1). Additionally, three SMC from dilution 10^–8^ were used as control. For accurate evaluation of the degradation capacities, the cultivation of the selected 21 SMC (18 SMC from dilution 10^–9^ and three SMC from dilution 10^–8^) was scaled up to 100 mL KM by 1:100 (v/v) ratio for 5 days (200 rpm, 24°C).

### Cell Density Measurement and Viable Cell Number Counting

Cultures in KM were left standing for 10 min on the bench at room temperature to allow sedimentation of large suspended keratin particles after cultivation. A total of 200 μL cell suspension was transferred to 96-well plate. Cell density was assessed by optical density (OD_600nm_) using a microplate reader (Biotek, ELx808).

To assess the viable cell number after serial dilutions from KMCG6, 200 μL diluted cell suspension was spread on LB agar plates. Cell numbers were counted according to observable colony forming units (CFU) on plates after 48 h growth at room temperature.

### Enzyme Activity Assays

#### Protease Activity Assay

Protease activity of microbial consortia was assessed with azocasein (Sigma-Aldrich, St. Louis, MO, United States) as described previously (Kang et al., 2018). 100 μL supernatant with 50 μL 1% (w/v) azocasein were incubated at 30°C for 30 min with shaking at 200 rpm, then stopped by adding 150 μL 10% (w/v) trichloroacetic acid and incubated at 4°C for 15 min. 100 μL mixture was mixed with 100 μL of 0.5 M NaOH after centrifugation. Absorbance was recorded in 96-well plates at 415 nm and one unit (U) of protease activity was defined as 0.01 increase in absorbance.

#### Keratinolytic Activity Assay

Preparation procedure for azokeratin and associated keratinolytic activity assay were described previously (Kang et al., 2018). The prepared keratin materials were coupled with a diazotized aryl amine to produce a chromophoric derivative, sulfanilic acid azokeratin. Keratinase activity of microbial consortia was assayed using azokeratin as a substrate. The reaction mixture of supernatant and azokeratin were incubated for 1 h at 30°C with shaking at 200 rpm, then cooled to room temperature for 5 min. 200 μL supernatant without azokeratin was transferred to 96-well plates and absorbance was measured. One unit (U) of keratinase activity was defined as the amount of enzyme required for a 0.01 increase in absorbance.

### Residual Keratin Substrate Weight

Keratin residue was collected from all of the microbial consortia after 5 days of cultivation in KM. The residual substrate was washed with deionized water using vacuum filtration and filter paper (Whatman, pore size: 8–12 μm) to remove microbial biomass. Washing was repeated until the flow-through was colorless. Biomass was then dried at 50°C for 48 h. The residual substrate was weighed and reported as the percentage (w/w%) of initial keratin substrate.

### Composition Analysis of the Simplified Microbial Consortia

A total of 5 mL cell suspension was collected from each SMC after 5 days of cultivation. DNA extraction was done using the FAST Soil DNA Kit (MP Biomedicals, United States), following the manufacturer’s instructions. PCR amplification and sequencing preparation were performed as previously described (Nunes et al., 2016), using the primers Uni341F (5′-CCTAYGGGRBGCASCAG-3′) and Uni806R (5′- GGACTACNNGGGTATCTAAT-3′) flanking the V3 and V4 regions of the 16S rRNA gene (Takai and Horikoshi, 2000; Klindworth et al., 2013). Purification of PCR products was done with Agencourt AMPure XP beads (Beckman Coulter Genomics, MA, United States) according to the manufacturer’s instructions. They were further quantified using Quant-iT High-Sensitivity DNA Assay Kit (Life Technologies) and pooled in equimolar concentrations using SequalPrep Normalization Plate (Thermo Fisher Scientific) before concentration using the DNA Clean and Concentrator-5 kit (Zymo Research, Irvine, CA, United States). Finally, a 20 pM pooled library was subjected to paired-end (2 × 250 bp) high-throughput sequencing on an Illumina MiSeq platform (Illumina, San Diego, CA, United States) using MiSeq reagent kit v2. Raw sequencing data were handled as previously described by respecting best practices guidelines (Schöler et al., 2017). The MiSeq Controller Software was used to perform the sequence demultiplexing and sequencing adapters and primers were trimmed using cutadapt v1.18 (Martin, 2011). Trimmed reads were then processed using a custom *BioDSL* pipeline^1^. Specifically, paired-end reads were merged and assembled pairs shorter than 300 bp or an average Phred score quality below 25 were discarded. Clean reads were clustered in OTU using a 97% sequence similarity threshold using *cluster_otus* and USEARCH v7.0.1090 and chimeric OTUs removed using UCHIME (Edgar et al., 2011). Each OTU cluster was given a taxonomic annotation using mothur with *classify.seq()* command (Schloss et al., 2009) and OTUs were assigned taxonomy using RDP Classifier (Wang et al., 2007) against the RDP database Trainset 9 (Cole et al., 2013). Representative OTU sequences were aligned against references using mothur and an approximate maximum likelihood phylogenetic tree was built using FastTree (Price et al., 2010). A read contingency table with OTU information including enrichment process was exported at the species level. A phylogenetic tree of KMCG6 was constructed based on OTU sequences using MEGAX (Kumar et al., 2018) by the maximum likelihood method with a bootstrap value of 1000 replications. Raw sequence data sets of 21 SMC are available at the NCBI Short Read Archive (SRA) with the BioProject ID: PRJNA562070.

### Strain Identification

Simplified microbial consortia from dilution 10^–9^ with high keratinolytic activity were plated on LB agar (24°C, 48 h). To secure strain purity for accurate identification and isolation, a single colony was picked and inoculated into LB medium for overnight cultivation until OD_600nm_ reached 0.7 – 0.8 (250 rpm, 24°C), then plated again. This procedure was repeated three times until all colonies on the LB plates had the same morphological characteristics. Afterward, one single colony was picked, cultured in LB medium overnight. DNA was extracted from 2 mL culture with FAST Soil DNA Kit (MP Biomedicals, United States), following the manufacturer’s instructions. The DNA was used as the template with primers 27F (5′-AGAGTTTGATCMTGGCTCAG-3′) and 1492R (5′-TACGGYTACCTTGTTACGACTT-3′) to amplify the full 16S rRNA gene, followed by Sanger sequencing (Russel et al., 2017). The obtained sequence was used as a query to do the sequence alignment and homology search with the assembled OTUs from KMCG6, which was performed by using BLAST with default parameters. This 16S rRNA gene sequence is available in the NCBI GenBank database (accession number: MN368255).

### Statistical Analysis

Euclidean distance was calculated to determine the functional dissimilarity of SMC from different dilutions in terms of cell density, enzymes activity, and residual ratio. The multivariate homogeneity of dilution group dispersion was analyzed with *betadisper* (R-package *vegan*) (Oksanen et al., 2010) and statistical differences was inferred by one-way ANOVA followed by *post hoc* Tukey’s HSD test. The significance level was defined as *p* < 0.05. For the selected 21 SMC, grouping was performed according to the compositional similarity using weighted UniFrac distance metric (Lozupone et al., 2011). Statistical differences of degrading capacities among SMC groups, KMCG6, and single strains were performed by one-way ANOVA using *post hoc* Tukey’s HSD test (*p* < 0.05). Relationships between microbes and degrading capacities were evaluated by using Pearson’s correlation with coefficients >|0.65| and visualized with *Gephi* (Bastian et al., 2009). All of the statistical analysis in this study was achieved in RGui software (version 3.5.0) (R Core Team, 2013).

## Results and Discussion

### Presentation of the Initial Microbial Consortium KMCG6

**FIGURE 2:**
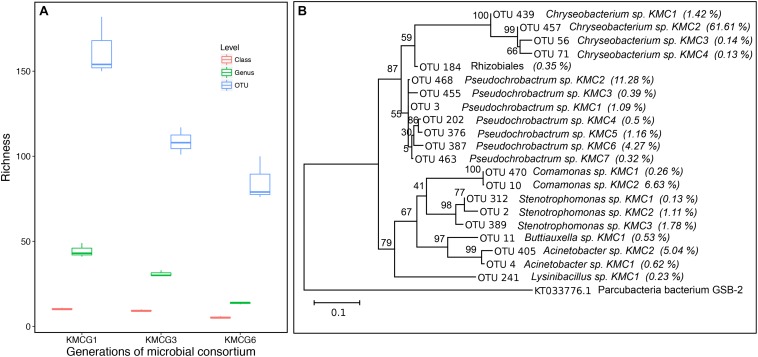
Microbial diversity through the enrichment process. **(A)** The observed richness values at class, genus, and OTU taxonomic levels from different generations during the enrichment process (KMCG1, KMCG3, and KMCG6). **(B)** Phylogenetic tree of dominant OTUs from KMCG6 (relative abundance >0.1%), Parcubacteria bacterium GSB-2 was set as the outgroup species. Bootstrap values are displayed at each node.

Microbial consortium KMCG6, displaying efficient keratinolytic activity, was obtained from Kang et.al using six successive generation batches as previously described (Kang et al., 2018). Taxonomic composition analysis (class, genus, and OTU) along the enrichment process was done at different generation batches (KMCG1, KMCG3, and KMCG6), showing a progressive decrease in consortia complexity (richness) along generation time (Figure 2A). Seven known genera (*Pseudochrobactrum*, *Chryseobacterium*, *Lysinibacillus*, *Acinetobacter*, *Buttiauxella*, *Stenotrophomonas*, *and Comamonas*) and one Rhizobiales were detected with a relative abundance >0.1% from KMCG6 (Figure 2B). KMCG6 comprised more than 21 dominating OTUs (>0.1%). Seven OTUs were classified to *Pseudochrobactrum*, which was the most diverse genus, followed by *Chryseobacterium* and *Stenotrophomonas*, both containing four OTUs. Notably, 61.61% of the total sequences were clustered to OTU_457, representing *Chryseobacterium* sp. KMC2 in KMCG6. Representatives from these three genera have been reported with keratinolytic activity toward feather keratin (Tamreihao et al., 2019; Yusuf et al., 2019). Nevertheless, the roles of these different microbes in KMCG6 were still not clear. Even cheaters are likely to be co-selected directly during the early stages of the enrichment (Jacquiod et al., 2013; Fredrickson, 2015). Therefore, simplification of KMCG6 was carried out in order to obtain potentially more controllable SMC for downstream applications. Additionally, a simplification of the community will make it easier to reveal and understand the individual roles of the strains in the consortia.

### Optimal Dilution for Construction of a Library of Simplified Microbial Consortia

**FIGURE 3:**
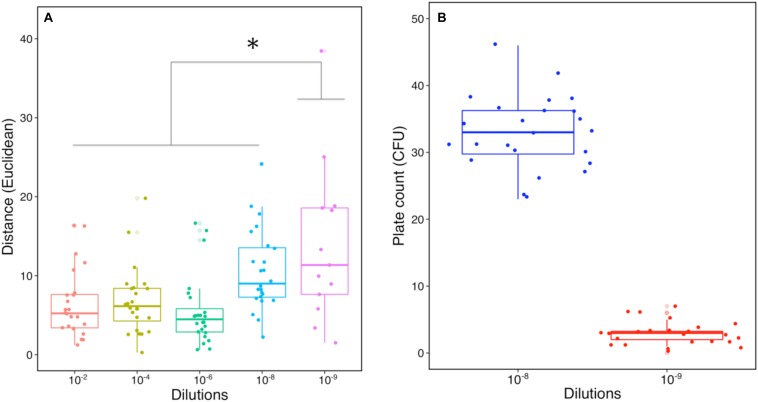
Characteristic comparison of SMC from the dilution-to-extinction culture. **(A)** Distance-based comparison using several characteristics including OD_600nm_, protease and keratinase activity (Euclidean distance, *n* = 24). Star indicates a significant statistical difference between dilutions with one-way ANOVA followed by *post hoc* Tukey’s HSD test (*p* < 0.05). **(B)** Numbers of CFU from dilution 10^–8^ and 10^–9^ by plate counting (*n* = 24).

Once an efficient pre-enriched consortium is secured, determining an optimal dilution is a critical step to obtain good functional heterogeneity in the subsequent SMC. Indeed, the extent of dilution and the corresponding reduction in microbial diversity were already shown to be not consistent during the dilution process of a soil microbial community (Philippot et al., 2013). Here we applied an integrated functional approach to select the optimal dilution, including measures of cell density, protease, and keratinase activity. Approximately 21% (5) and 92% (22) of the diluted replicates from dilution 10^–9^ and 10^–10^ no longer displayed cell growth in KM, respectively. Hence, dilution 10^–10^ was excluded based on a lack of growth and activity. Comparisons of different dilutions were made according to their characteristics of degrading capacities (cell density and enzyme activities) with Euclidean distance (Figure 3A). The profiles of dilution 10^–2^ to 10^–8^ had no visible difference, whereas dilution 10^–9^ showed significantly higher variability (*p* < 0.05) compared to other dilutions. The mean number of CFU in dilution 10^–8^ was 33 and dilution 10^–9^ was three, showing an expected decrease of 10-fold (Figure 3B). The increased variability and CFU indicated good potential for assembling effective SMC from dilution 10^–9^. Therefore, dilution 10^–9^ was used to further construct a library containing 96 SMC (Figure 1 and Supplementary Figure S1). Previous studies showed that few replicates cultured from distinct dilutions resulted in a limited heterogeneity of the functional microbial consortia (Ho et al., 2012a, b), making statistical conclusions nor very reliable for supporting the selection of optimal dilution and desired SMC candidates. Therefore, we decided to upscale the number for better statistical representation, leading to generate enough SMC with heterogeneity/variability in terms of diversity and functions. This can contribute to obtaining the promising SMC for keratin degradation. Certainly, a parsimonious approach ought to rely on an adjusted number from prior evaluation of the taxonomic composition and abundance of the members in the pre-enriched community.

### Diversity and Structure of Simplified Microbial Consortia During Keratin Degradation

**FIGURE 4:**
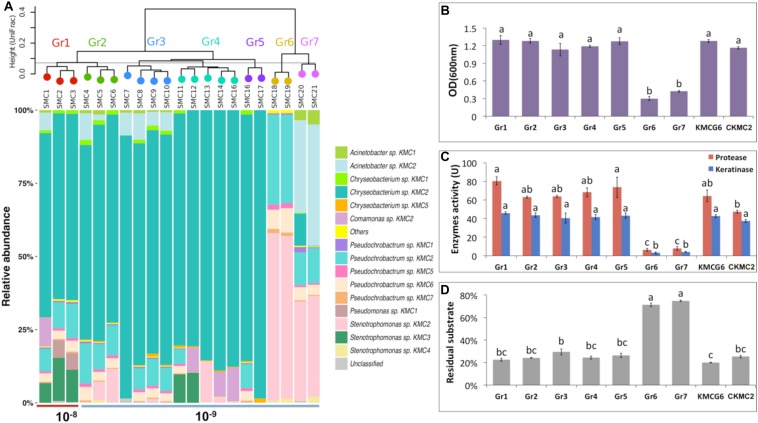
Integrated comparison of selected SMC. **(A)** 21 SMC divided into seven groups based on their community similarities from 16S rRNA gene analysis (weighted UniFrac distance). Group 1 (Gr1) includes three simplified microbial consortia SMC1, SMC2, and SMC3 from dilution 10^–8^. A total of 18 SMC (SMC4 – SMC21) were clustered into six groups (Gr2 – Gr7) from dilution 10^–9^. **(B)** Cell density (OD_600nm_) comparison of group 1–7, KMCG6 and the most abundant species in the initial KMCG6, *Chryseobacterium* sp. KMC2 (CKMC2). **(C)** Comparison of enzymes (protease and keratinase) activity. **(D)** Comparison of the residual substrate ratio. Lowercase letters (e.g., a, b, and c) in **(B–D)** refer to significant differences between groups with one-way ANOVA followed by *post hoc* Tukey’s HSD test (*p* < 0.05).

The taxonomic classification of 21 SMC was investigated at the OTU level using 16S rRNA gene amplicon sequencing (Figure 4A). In total, 15 OTUs were found in the SMC with a relative abundance above 0.1%, including 12 OTUs that were also observed in KMCG6. The three new detected members were *Chryseobacterium* sp. KMC5, *Pseudomonas* sp. KMC1 and *Stenotrophomonas* sp. KMC4. The relative abundances of these three OTUs were below 0.1% in KMCG6, suggesting that these initial rare species could be enriched during our SMC construction procedure. Recent studies have demonstrated that rare taxa can play essential roles in community functioning and stability (Jia et al., 2018). Hence, the functions of these rare species in keratin degradation could be investigated from the SMC in the library. SMC were clustered into seven groups according to their taxonomic composition (weighted UniFrac). Three SMC (SMC1, SMC2, and SMC3) from dilution 10^–8^ clustered as group 1 (Gr1), all featuring the dominant *Chryseobacterium* sp. KMC2 (>62.9%), which initially constituted the majority of KMCG6. Members of *Chryseobacterium* have previously been isolated and identified from different environments when keratin was supplied as the carbon source (Lloyd-Jones et al., 2010; Herzog et al., 2016). Relative abundances of *Stenotrophomonas* sp. KMC3 and *Pseudochrobactrum* sp. KMC2 were above 5% in Gr1. As expected, the composition of SMC in dilution 10^–9^ was set apart from 10^–8^, showing a substantial difference between these two dilutions. Likewise, previous studies showed that the dilution procedure had an impact on the biodiversity of microbial community (Yan et al., 2015; Stadler et al., 2018). Interestingly, 18 SMC from dilution 10^–9^ were divided into six groups (Gr2 – Gr7) with heterogeneous profiles in terms of OTU diversity (OTUs = 2–12), demonstrating the heterogeneity of diversity generated by this approach. 14 SMC (SMC4 – SMC17) from dilution 10^–9^ still featured the dominant *Chryseobacterium* sp. KMC2. Four SMC (SMC18 – SMC21) contained little to no *Chryseobacterium* sp. KMC2 and they were classified into two different groups (Gr6 and Gr7). It revealed that the diversity indices of SMC with a highly variable were not only from different dilutions, which also could obtain from the same dilution.

### Comparative Analysis of Keratin Degradation Capacities

The initial microbial community is likely to be divided into different functional groups along with the serial dilution process due to random reassembly of microbes caused by extinction and sampling effects (Roger et al., 2016). Keratinolytic characteristics of SMC were measured, which were summarized according to the taxonomic grouping of SMC (Figures 4B–D). Additionally, the dominant species *Chryseobacterium* sp. KMC2 was isolated and identified by 16S rRNA gene sequencing. The degradative capacities of this single species and KMCG6 were compared to these groups. Two distinct keratinolytic performance categories were present among the groups in terms of cell density, enzyme activities, and residual substrate ratio. The first category includes mostly groups from dilution 10^–9^: Gr2, Gr3, Gr4, and Gr5, together with Gr1 (10^–8^), all displayed a good capability to degrade keratinous materials similar to that of KMCG6. This also verified that the taxonomic composition across microbial communities was variable, while still exhibiting a stable functional profile (Louca et al., 2017). Gr6 and Gr7 had poor performances with weak keratinolytic activity.

All SMC from 10^–8^ (SMC1, SMC2, and SMC3) showed similar cell density, with OD_600nm_ reaching up to (1.15 – 1.3). The OD_600nm_ of the 18 SMC from dilution 10^–9^ ranges between 0.1 and 1.2. No visible difference in biomass generation was observed between the good SMC performers and the initial KMCG6. It is worth noticing that the protease activities from Gr1 and Gr5 consortia were significantly higher than the pure culture of *Chryseobacterium* sp. KMC2. A previous study showed that the co-culture of two *Bacillus* strains exhibited remarkable hydrolases activities and enhanced the yield of surfactin using distillers’ grains as a carbon source (Zhi et al., 2017). Thus, it suggested that Gr1 and Gr5 consortia had good degradation efficiency in terms of proteolysis, which can be utilized for keratin valorization. When considering the keratin residue ratio, SMC from dilution 10^–9^ had very divergent values, indicating that we succeeded in generating heterogeneous functional SMC from KMCG6. More importantly, well-performing consortia with reduced diversity maintained equal degradative capacities compared to KMCG6. Top-down enrichment-based methodology is considered as an effective way to get microbial consortia for biomass degradation (Gilmore et al., 2019). We further explored the optimization procedure to obtain SMC. This illustrated that the dilution-to-extinction strategy is an alternative way to obtain optimal candidates with stable (or even enhanced) degradation capabilities while harboring a reduced complexity.

### Potential Biotic Interactions Inferred From Correlation Network

**FIGURE 5:**
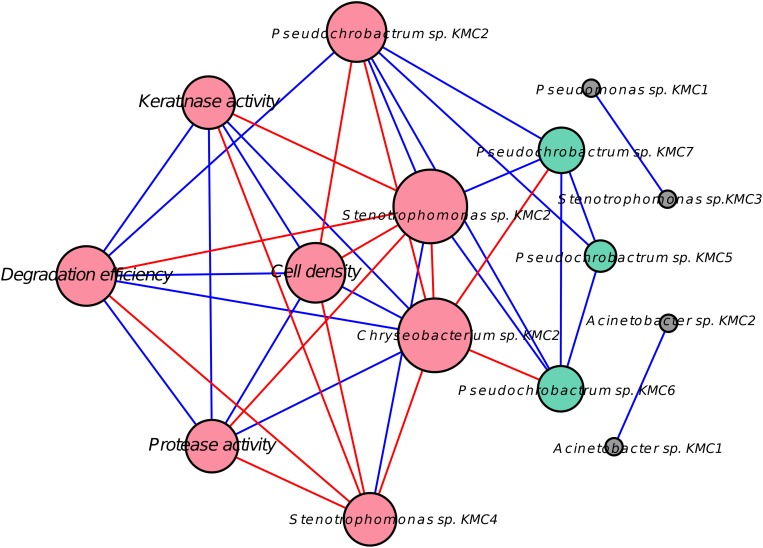
Network showing relationships between species and degradative capacity using Pearson’s correlation node. The represent either OTUs or characteristics of keratin degradation, e.g., degradation efficiency and keratinase activity. Red nodes indicate the direct correlation with degradation efficiency (DE). Green nodes had an indirect correlation with DE through other species. Gray nodes had no significant correlation with DE. The size of the nodes reflects the number of connections (degree); larger nodes have more significant connections. Blue lines represent positive correlations and red lines represent the negative correlation.

An increasing number of methods have been developed to unravel microbial interactions in microbial communities, including local similarity analysis (Ruan et al., 2006) and similarity-based techniques, mainly using Pearson’s or Spearman’s ranked correlations, for abundance data (Faust and Raes, 2012). To enhance the association between species and degradative capacity, a Pearson’s correlation-based network was adopted to screen for potential biotic interactions (Figure 5). As expected, the cell density and enzyme activities had a significant positive correlation with degradation efficiency. Seven species (*Chryseobacterium* sp. KMC2, *Pseudochrobactrum* sp. KMC2, *Pseudochrobactrum* sp. KMC5, *Pseudochrobactrum* sp. KMC6, *Pseudochrobactrum* sp. KMC7, *Stenotrophomonas* sp. KMC2, and *Stenotrophomonas* sp. KMC4) were connected with keratinolytic activity, which are likely to be key players in the keratin degradation. Keystone taxa are thought to drive the structure and functionality of microbial communities (Banerjee et al., 2018). Among them, the dominant *Chryseobacterium* sp. KMC2 was the only species that had positive correlations with all of the keratinolytic characteristics, suggesting that *Chryseobacterium* sp. KMC2 is the keystone species in the degradative process. Interestingly, all of the other species in this correlated network have negative correlations with *Chryseobacterium* sp. KMC2. Negative correlations were deemed to dominate interactions among culturable microbial species (Foster and Bell, 2012), likely due to a selection bias toward strong competitors displaying fast growth and efficient use of resources, thus leading to fierce competition and competitive exclusion processes. The effect of these negative correlations may promote stability in SMC. *Pseudochrobactrum* sp. KMC2 has a positive correlation with the degradation efficiency. Meanwhile, it has numerous positive correlations with other species, such as *Stenotrophomonas* sp. KMC2 and *Pseudochrobactrum* sp. KMC7. *Stenotrophomonas* sp. KMC2 is associated with a decrease in degradation efficiency, showing that it could benefit from *Pseudochrobactrum* sp. KMC2. Four species (*Acinetobacter* sp. KMC1, *Acinetobacter* sp. KMC2, *Stenotrophomonas* sp. KMC3, and *Pseudomonas* sp. KMC1) only had satellite correlations. Although no direct link to keratinolytic activity was seen with these four species, the fact that they remain “rare” in terms of sequence abundance could explain the lack of correlation due to a resolution issue. Nevertheless, species with relatively low abundances can become more prevalent following environmental change (Jia et al., 2018). Further experimental evidence is necessary to clarify their roles in keratin degradation. It showed that network analysis based on correlations from selected SMC could be used to infer potential microbial interaction at the species level.

In conclusion, we have demonstrated that dilution-to-extinction represents an efficient strategy to assemble SMC involved in recalcitrant material degradation (Figure 1). A library of functional microbial consortia was constructed after determining the optimal dilution. The SMC were assessed by taxonomic analysis and keratinolytic capacities evaluation. Several potentially interesting candidates were retrieved, all still displaying efficient keratinous material degradation capabilities without losing crucial enzymatic activities. Additionally, potential biotic interactions among microbes were evaluated using a correlation network. This approach revealed that obtaining the simplified functional consortia from high microbial diversity environments is feasible, and also point toward further options for optimization and designing microbial consortia.

## Data Availability Statement

The datasets generated for this study can be found in the NCBI BioProject ID: PRJNA562070, NCBI GenBank database accession number: MN368255.

## Author Contributions

DK and SS contributed to the conception of this study. DK, SJ, and JH contributed to the development of the research plan. DK performed all the experiments and wrote the first draft of the manuscript. SJ, JH, and JN contributed to revising the manuscript. DK, SW, and JN conducted all the data analyses. SS was responsible for the integrity of the work and overall supervision. All authors contributed to the interpretation of data and approved the manuscript.

## Conflict of Interest

The authors declare that the research was conducted in the absence of any commercial or financial relationships that could be construed as a potential conflict of interest.

## Footnotes

1 http://maasha.github.io/BioDSL/
