# Supplementary material for: Construction of Simplified Microbial Consortia to Degrade Recalcitrant Materials Based on Enrichment and Dilution-to-Extinction Cultures
Source: Front Microbiol. 2020 Jan 10;10:3010. doi: 10.3389/fmicb.2019.03010 (PMC6968696; doi:10.3389/fmicb.2019.03010)
Supplement: Supplementary file 1 [file Data_Sheet_1.pdf]

Supplemental Material for

Construction of simplified microbial consortia to degrade recalcitrant materials based on enrichment and dilution-to-extinction cultures

Dingrong Kang[1], Samuel Jacquiod[1,2], Jakob Herschend[1], Shaodong Wei[1], Joseph Nesme[1], Søren J. Sørensen[1]*

[1]Section of Microbiology, Department of Biology, University of Copenhagen, Copenhagen, Denmark

[2]Agroécologie, AgroSup Dijon, INRA, Univ. Bourgogne Franche-Comté, France

*Corresponding author

Supplementary data includes 1 figure.

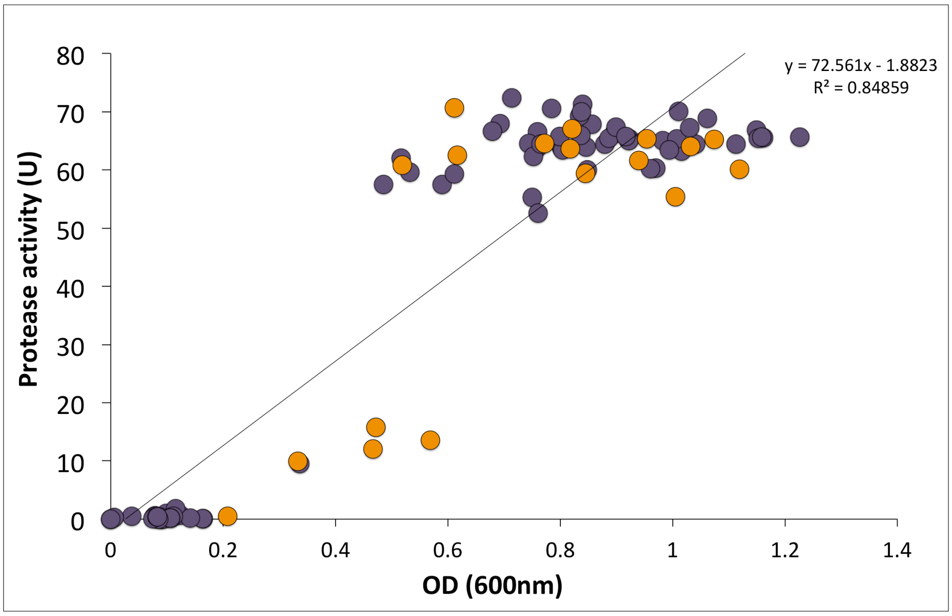

Fig. S1. The biological characteristics (cell density and protease activity) of 96 simplified microbial consortia (SMC) from dilution 10^{-9}. Dots represent each of the 96 biological replicates, including the 18 simplified microbial consortia (yellow dots), which were selected for further functional assessment.
